# Monoaminergic Modulation of Learning and Cognitive Function in the Prefrontal Cortex

**DOI:** 10.3390/brainsci14090902

**Published:** 2024-09-06

**Authors:** Natalie Boyle, Sarah Betts, Hui Lu

**Affiliations:** Department of Pharmacology and Physiology, School of Medicine and Health Sciences, The George Washington University, Washington, DC 20037, USA; nboyle1@gwu.edu (N.B.); sarah.betts@gwmail.gwu.edu (S.B.)

**Keywords:** monoamines, cognition, prefrontal cortex, dopamine, serotonin, norepinephrine, learning

## Abstract

Extensive research has shed light on the cellular and functional underpinnings of higher cognition as influenced by the prefrontal cortex. Neurotransmitters act as key regulatory molecules within the PFC to assist with synchronizing cognitive state and arousal levels. The monoamine family of neurotransmitters, including dopamine, serotonin, and norepinephrine, play multifaceted roles in the cognitive processes behind learning and memory. The present review explores the organization and signaling patterns of monoamines within the PFC, as well as elucidates the numerous roles played by monoamines in learning and higher cognitive function.

## 1. Introduction

The prefrontal cortex (PFC) exists as a pivotal brain region orchestrating higher cognitive functions and executive control, including the processes of learning and memory [1,2,3]. Integral to this function is the PFC’s ability to integrate information from diverse sensory modalities, thereby facilitating executive functioning. Extensive reciprocal connections with various regions, including the amygdala, hypothalamus, midbrain, and neuromodulatory systems allow for the assimilation of various sensory modalities and for subsequent PFC top-down control [1]. 

Structurally, the PFC in rodents lacks the granular layer (layer IV) characteristic of the primate PFC, making it entirely agranular [4]. Despite this, the rodent PFC can be parcellated into distinct regions. Efforts to map the mouse PFC have revealed variances in nomenclature and anatomical boundaries between different atlases, but commonly recognized regions include the infralimbic, prelimbic, and medial orbital cortices [5]. These areas show distinct patterns of connectivity and cytoarchitecture, although the granular layer IV is absent. The medial and orbital regions in rodents receive projections from the mediodorsal (MD) thalamus, similar to primates, though the MD does not target dorsolateral areas in rodents as it does in primates [4,5]. In primates, the dorsolateral prefrontal cortex (dlPFC) is the crucial region for working memory (WM), decision-making, and higher-order cognitive functions. The dlPFC supports the active retention and processing of task-relevant information, facilitating complex behaviors and future-oriented actions. This region is particularly studied using delayed-response tasks, which reveal its pivotal role in maintaining and manipulating information over short periods [4].

Primates possess a more differentiated and layered PFC, enabling more complex cognitive abilities, whereas rodents exhibit a more uniform agranular structure but still perform analogous cognitive tasks. These differences could hinder translation of data between species and must be considered; however, PFC studies from a variety of species including rodents can be useful to overall understanding [5]. Despite the structural differences of primates’ more differentiated and layered PFC and rodents’ uniform agranular structure, prefrontal functions, especially working memory and executive control, and PFC-mediated behaviors are similar across the species [5].

Research has demonstrated robust activation of the medial PFC (mPFC) during working memory tasks, while lesion studies underscore the indispensable role of the mPFC in learning and memory processes [6,7,8]. Electrophysiological investigations have further unveiled the presence of neurons within the mPFC that exhibit selective responsiveness to spatial cues and are capable of encoding object location, a crucial component for the maintenance of spatial information during working memory tasks [9,10,11]. 

Working memory in the prefrontal cortex relies on the interaction of pyramidal cells via AMPA and NMDA receptor synapses situated on dendritic spines [12]. As glutamate is released, it binds to AMPA receptors, allowing Na^+^ to enter and depolarize the postsynaptic membrane in an additive manner [13]. If the depolarization is sufficient, NMDA receptors will open, allowing both calcium ions and sodium ions to pass through the postsynaptic membrane [13]. NMDA receptors with the NR2B subunit are kinetically slow, allowing persistent activity to occur in the PFC in the absence of stimulation, thus forming the basis of working memory [12]. This process is further modulated by neurotransmitters to synchronize cognitive state with arousal levels [9,14]. Research has found overexpression of the NR2B subunit improves rodent performance in a series of working memory tasks and enhances prefrontal cortical long-term potentiation without altering long-term depression [15]. The firing pattern induced by NR2B is fundamentally different than that of long-term memory consolidation, where instead architectural changes, such as insertion of AMPAR into the post-synaptic density, store events and regulate strength of synaptic reactivity [12,16,17,18].

The PFC is composed of ~75–80% glutamatergic projection neurons and ~20–25% GABAergic local circuit interneurons [19]. The synergistic local activity of these excitatory and inhibitory neurons across layers I–V is essential for coordinating and maintaining PFC network dynamics [20,21,22]. These dynamic circuits are profoundly influenced by various neuromodulatory systems, as the PFC exhibits reciprocal connectivity with many of the key monoamine systems. Mutual connectivity between the PFC and the midbrain dopamine system, LC-norepinephrine system, and serotonergic systems each play a critical role in modulating PFC functioning, encompassing diverse aspects of learning and working memory [19]. In the forthcoming review, we aim to explore the multifaceted roles of select neuromodulators linked to the PFC and elucidate their overarching contributions to the learning and memory processes within this region.

## 2. Monoamines and the Prefrontal Cortex: A Comprehensive Overview

**Figure 1:**
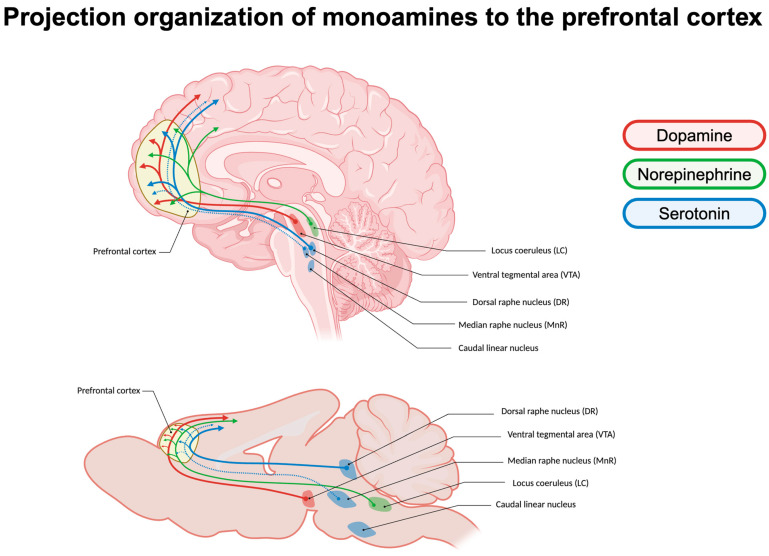
Organization of monoamine projections to the prefrontal cortex. Key centers of monoamine production that project to the PFC in both humans (**top**) and rodents (**bottom**). Dopamine (red) includes the ventral tegmental area (VTA). Norepinephrine (green) includes the locus coeruleus (LC). Serotonin (blue) includes the dorsal raphe nucleus (DR) and median raphe nucleus (MnR). The MnR projection to the PFC is secondary to the DR, indicated by a dotted arrow.

The term “monoamines” refers to a group of neurotransmitters characterized by an amino group linked to an aromatic ring via a two-carbon chain [23]. The PFC in both the human and rodent brain is innervated by projections from monoaminergic nuclei originating in the brainstem, including the dorsal and median raphe nuclei, locus coeruleus, and ventral tegmental area (Figure 1) [19]. Monoaminergic systems are critical components for maintaining various higher cognitive processes, including arousal, working memory, and emotional responses [23]. Due to their notable role in these higher order PFC functions, it is not surprising that many monoamine receptors are the pharmacologic targets for several psychiatric disorders, including depression, schizophrenia, and anxiety [19,24]. The tight-knit relationship between monoamines and the prefrontal cortex is critical for crucial higher cognitive functions. Three major monoamines, dopamine, serotonin, and norepinephrine, exert profound influences on PFC cognitive function through their interactions with specific receptors distributed throughout this brain region. 

### 2.1. Projection Organization of Monoamines in the Prefrontal Cortex

#### 2.1.1. Dopamine Signaling

Dopamine (DA) plays a pivotal role in regulating various cognitive functions under the purview of the prefrontal cortex (PFC), including attention, working memory, decision-making, and goal-directed behavior [9,25,26]. In order to influence various motivational processes and encode rewards or aversion, dopamine is transmitted to the PFC through the mesocortical pathway originating from the ventral tegmental area (VTA) [27,28,29,30]. VTA-DA projecting neurons convey signals to the PFC through two modes: tonic firing, characterized by a steady, pacemaker-like rate, and phasic firing, marked by transient bursts of high frequency [31]. In the prefrontal cortex, dopamine signaling is further influenced by the low density of dopamine transporters (DATs). This low DAT expression leads to reduced dopamine reuptake, resulting in greater diffusion distances and a more widespread and prolonged signaling effect. Consequently, DA transmission in the prefrontal cortex is less tightly controlled compared to other brain regions, such as the striatum, where DAT density is higher [32,33,34]. Due to the scarcity of DATs, dopamine reuptake in the PFC is primarily handled by the norepinephrine transporter (NET), which has a higher affinity for dopamine than the DAT [35]. In rats, blocking NETs or α2-adrenoceptors with antagonists such as atipamezole (3 mg/kg, IP) significantly increases both NE and DA levels in the mPFC, suggesting DA release in the mPFC is intricately regulated by the NE system [36,37]. In the deeper layers of the PFC, dopamine transmission is instead modulated by extraneuronal metabolism [38,39]. 

#### 2.1.2. Serotonin Signaling

Serotonin (5-HT) is a key regulator of both the central and peripheral nervous systems, influencing developmental signals and peripheral functions including appetite, mood, platelet aggregation, and vasoconstriction [40,41,42,43]. In the CNS, serotonin is critical for flexible responding, especially at the level of changing stimulus–reward contingencies, plasticity, and rate of learning. Serotonin is synthesized in the raphe nuclei in the midbrain, and serotonergic innervation throughout the brain matures early in the primate postnatal brain [44,45]. The rostral nuclei, including the caudal linear nucleus, dorsal raphe nucleus (DR), and median raphe nucleus (MnR), project to forebrain areas and contain the majority of serotonergic neurons in the brain [46]. The PFC receives its densest serotonergic innervation from the DR with secondary input from the MnR [46]. In the PFC, axons from the DR and MnR influence both pre- and postsynaptic receptors [44,45,47]. The majority of DR serotonin neurons exhibit a steady pace-maker-like release of serotonin, and reuptake is handled by the serotonin reuptake transporter (SERT) [46,48]. Prefrontal afferents additionally send projections back to the dorsal raphe, allowing for controlled regulation of 5-HT neurons [49]. 

#### 2.1.3. Norepinephrine Signaling

Norepinephrine (NE) plays an important role in arousal, attention, and learning [50]. NE axons stem from the pontine locus coeruleus (LC) to release NE throughout the cerebral cortex, with innervation remaining densest in frontal cortical areas, primarily the PFC [50,51,52,53]. Reciprocal projections from the PFC to LC are both directly excitatory or inhibitory via local interneurons [54,55]. Notably, LC neurons projecting to the PFC are physiologically distinct from those projecting to alternate regions, such as the primary motor cortex, firing at a rate three-fold faster to the PFC [56]. 

### 2.2. Cellular Distribution of Monoamine Receptors in the Prefrontal Cortex

#### 2.2.1. Dopamine Receptors

Upon release in the PFC, dopamine binds to five different classes of G-protein coupled receptors, namely D1, D2, D3, D4, and D5 [57]. These receptors are broadly classified into the ‘D1-like’ family (G_s_ coupled), or the ‘D2-like’ family (G_i/o_ coupled), based on structural and pharmacological similarities [57,58,59]. Within the layers of the prefrontal cortex, the D1R-family is more highly expressed than the D2R-family, and is located on both pyramidal neurons and GABAergic interneurons [58,60,61]. Among the D1R-family, the D1 and D5 subtypes demonstrate a partially overlapping distribution in the PFC [62]. D5 receptors are localized on a subset of dendritic spines of pyramidal neurons and terminals of GABAergic interneurons where they coexist with D1 receptors [59,62]. In other spines and terminals, only D1 receptors are present [59,62]. Notably, layers V and VI of the cortex contain the highest density expression of both D1R and D2R families [63,64]. Typically, the D1R family (D1 and D5) induces excitation, while the D2R family (D2, D3, D4) inhibits neuronal response, reflecting opposing actions [65]. 

#### 2.2.2. Serotonin Receptors

Over the course of development, there are extensive changes in the expression levels of several serotonin receptors in the PFC [44]. Current work has identified seven major families of serotonin receptors, with multiple subtypes within each family [44,47]. With the exception of 5-HT3, all serotonin receptors are G-protein coupled, each with molecular and structural differences that alter the physiological response in corresponding neurons [44,66]. The 5-HT1 and 5-HT5 families are G_i/o_ coupled, and binding typically leads to inhibition of neuronal responses [44]. The G_q_ coupled (5-HT2) and G_s_ coupled (5-HT4, 5-HT6, and 5-HT7) families act with a similar mechanism to one another; binding of 5-HT results in a decrease of potassium current and subsequent increase of nonselective cation current, causing local excitation [44,67,68]. 5-HT1A and 5-HT2A are the most well-studied families and are predominantly expressed on pyramidal neurons (70%) and some interneurons (30%) of the PFC [69]. Specifically, 5-HT1A receptors are densely expressed on the axon initial segment of pyramidal neurons, whereas 5-HT2A receptors are largely located in apical dendrites [70]. The location of receptors allows for 5-HT to subsequently suppress action potential generation or amplify excitatory currents, respectively [70]. 

#### 2.2.3. Norepinephrine Receptors 

There are three major families of NE receptors that have been further categorized into subtypes: the α1 family (G_q_ coupled; α1A, α1B, α1D), α2 family (G_i/o_ coupled; α2A, α2B, α2C), and β (G_s_ coupled; β1, β2, β3) receptor families [52,71]. Each of the receptor classes are expressed in the PFC at different levels. α1 and α2 receptor families are highly concentrated in more superficial layers of the cortex (L1no-3a), while β1/β2 receptors are concentrated in the intermediate layers of the cortex (IIIb, IV) [72]. In the rodent PFC, all NE receptor subtypes are found localized presynaptically on noradrenergic axons, while the α2 receptor family is further found on postsynaptic neurons [73]. Of the α2 family, the α2A subtype expression is densest in the PFC and maintains the highest affinity for NE binding [74,75,76]. Similar to 5-HT and DA, engaging different NE receptors in the PFC has varying effects on the circuitry. For example, activation of α1-adrenergic subtypes tends to oppose the effects of β-adrenergic subtypes in the prefrontal cortex; the α1 family increases excitatory transmission, while the β family decreases glutamate-induced excitatory postsynaptic potentials (EPSPs) [77]. 

## 3. Monoamines and Behavior: Roles in Learning and Cognition

### 3.1. Dopamine: Reinforcement and Rule Coding

**Figure 2:**
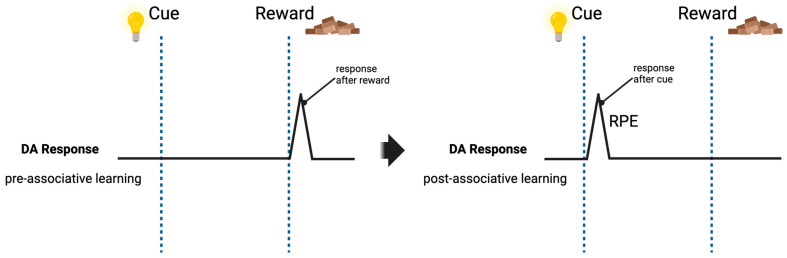
Dopamine and the reward prediction error (RPE). Studies have shown when a cue is first presented (light) followed by a reward stimulus (food), DA phasic burst response occurs at presentation of the reward. Following reinforcement learning, DA phasic burst activity shifts to the time of the cue presentation (light), encoding the reward prediction error.

Mounting evidence underscores the pivotal role of dopamine in learning and motivational control, particularly in associative learning and memory processes. As previously mentioned, dopaminergic projections to the PFC display distinct firing patterns: tonic and phasic firing. Dopaminergic phasic burst activity has been associated with the attribution of incentive salience to reward cues during associative learning. These associative circuits modulated by dopamine are sensitive to the relative timing and order of events, both of which are necessary to accurately predict rewarding or punishing reinforcement [78]. It is important to note that phasic dopamine transients, while critical for associative learning, do not themselves represent valence. Rather, they are crucial for the recognition of behaviorally relevant events throughout learning [79]. For example, when presented with a cue followed by an appetitive stimulus, dopaminergic neurons respond with short, phasic bursts [58]. Once animals successfully associate an unconditioned stimulus (cue) with a reward, phasic burst activation shifts from the time the appetitive stimuli is presented to the time of the cue [80]. These phasic changes in dopamine are hypothesized to encode the reward prediction error (RPE), or the difference between an expected reward following an action and the actual reward received (Figure 2) [81,82,83]. The RPE is an essential feature of reinforcement learning, allowing for one’s behavior to adapt to changing environments [81]. 

Furthermore, dopamine neuron activity varies significantly with age, impacting associative circuits. Dopamine activity throughout the lifespan follows an inverted-U shape curve, with peak activity occurring around puberty [84]. Elevated DA neuron activity during adolescence is linked to a heightened vulnerability to drug addiction, with previous studies showing increased VTA-DA firing is linked with self-administration of cocaine, sensitivity to lower doses of cocaine, and greater escalation of cocaine intake [84,85]. This vulnerability to drug addiction during adolescence underscores the critical role of heightened dopaminergic signaling in amplifying the incentive salience of drug-related cues, thereby facilitating strong associative memories.

This sensitivity to temporal dynamics extends beyond reward learning paradigms, as dopamine also plays a crucial role in fear learning processes. In fear learning paradigms, dopamine enhances the signal-to-noise ratio of responses to aversive stimuli, particularly in PFC neurons projecting to the dorsal periaqueductal gray, a critical area governing defensive behaviors [31]. Moreover, dopamine depletion is found to impair adaptive exploratory responses in fear-inducing environments, highlighting its role in associative fear learning [86].

In addition to reinforcement and aversive learning, the dopamine-mediated RPE drives social reinforcement learning. Social reinforcement involves learning the value of stimuli and actions from others rather than through individual experience, tying together reward and social learning. Naive gerbils observing a skilled demonstrator performing an auditory discrimination task for a reward displayed significantly decreased time to learn the task, as compared to unrewarded observers [87]. Blocking the gerbil’s D1R family receptors with SCH-23390 resulted in significant impairments in performance during the exposure session [87]. Additional human studies point to the impairment of social learning through the D2 receptor antagonist haloperidol (2.5 mg, capsule), particularly when social learning is the primary source of learning [88]. Moreover, dopaminergic activity rooted in the mesocortical pathway is modulated by social interactions. An increase in VTA-DA neuron firing is observed when mice interact with an unfamiliar conspecific, to which the strength of activation is correlated with rodent social hierarchy [89]. When entering a new social situation and establishing a previously learned hierarchy, dominant mice display stronger excitatory synaptic strength onto D1R neurons in the prefrontal cortex. Alongside dominant mice displaying higher D1R activation, “submissive” mice will instead have higher neuronal excitability in D2R-expressing neurons, which tend to exert inhibitory responses [90]. These contrasting yet complementary roles of D1R and D2R activation in the prefrontal cortex manifest throughout numerous aspects of learning and memory.

The interplay between D1R and D2R family activation in the PFC shapes learning processes across various domains. Both receptor subtypes contribute to rule coding in PFC neurons, albeit through distinct mechanisms. In both rodent and primate studies, when tasked with learning rule-based decision making, D1R and D2R in the PFC are found to make complementary modulatory contributions to mediate behavior [26,91]. These opposing responses are temporally sensitive and can help to regulate behavior and neural plasticity [78]. This coincides with evidence that the PFC is dependent on an optimum range of DA activity; for example, too little or too much D1R stimulation has detrimental effects on working memory [92,93]. The nonlinear relationship between DA levels and cognitive functioning is well known, highlighted by the inverted-U shape theory [94,95]. This theory suggests that both too little and too much DA can impair performance, and the subsequent degree of cognitive impairment or improvement varies depending on baseline DA levels and task [94,95,96]. For example, administration of a D1R agonist SKF81297 in the mPFC of monkeys during a working memory task resulted in dose-dependent consequences. Moderate levels of suppression lead to an enhancement in spatial tuning (15 nA), whereas higher levels of stimulation (40 nA) caused impairment in the working memory task [97]. Additionally, a previous review on the dorsolateral PFC (dlPFC) in primates demonstrated that the optimal dopamine state varies depending on the specific cognitive task [98]. Tasks that benefit from broad network connections, such as creative thinking, are supported by a neurochemical state with strong α2A-adrenergic receptor activation and lower dopamine D1 receptor activity. Conversely, tasks requiring precision and focus, such as solving math problems, benefit from higher dopamine D1 receptor activity. Thus, it is crucial to maintain appropriate regulation of dopaminergic activity in the PFC in order to properly orchestrate learning and memory dynamics. 

In recent years, the potential for cognitive enhancement through pharmacological modulation of dopamine levels in the PFC has garnered significant interest. The inverted-U functional effect of dopamine in the PFC suggests both insufficient and excessive DA levels impair cognitive performance, leading to the development of several compounds aimed at modulating dopamine levels to improve cognitive function. Many of these compounds, including CE-158, CE-123, and Sy-phenylpiracetam, work by inhibiting the dopamine transporter (DAT) to promote behavioral flexibility and cognitive enhancement in the PFC [99,100,101,102]. Other compounds, such as d-Govadine (d-GOV), increase glutamate excitation of VTA dopaminergic neurons [103,104]. This enables d-GOV to increase dopamine (DA) efflux in the mPFC without affecting the nucleus accumbens, distinguishing its pharmacology from many other dopaminergic drugs and likely contributing to its cognitive effects [105]. In a study exploring the potential of augmenting dopamine signaling as a therapeutic strategy to ameliorate cognitive impairments induced by compromised PFC GABA function, male rats were administered a GABAa antagonist, bicuculline methbromide (50 ng in 0.5 μL), via intraperitoneal injection for four days, which impaired their performance on a delayed-response working memory task. Treatment with d-GOV (dose) was found to significantly improve performance on the delayed-response working memory task in these rats, whose performance had been impaired by PFC GABAa antagonism [104].

### 3.2. Serotonin: Aversive Learning, Cognitive Flexibility, and Social Reward Prediction

Serotonin is a neuromodulator linked to both emotional and motivational aspects of behavior. A wide array of pharmacological treatments target 5-HT for various mood disorders, including depression, anxiety, and OCD. Emotional learning and emotional motivation, such as in the case of aversion, are therefore highly influenced by 5-HT. In human subjects, fMRI studies indicate lowering 5-HT impairs aversive Pavlovian learning, coinciding with reduced prediction-error signals in the orbitofrontal cortex [106]. In 5-HT knock-out studies, the IL-region of the rodent PFC displays significant morphological abnormalities as well as significant deficits in extinction recall following a Pavlovian fear conditioning paradigm [107]. 

A substantial body of research implicates serotonin’s involvement in cognitive flexibility, plasticity, and rate of learning. The ability to rapidly adjust to changes in the environment is not a static process and requires flexible adaptation during decision-making and learning processes. Mice are found to demonstrate variable behavioral flexibility during decision making modulated by 5-HT activity [108]. These signals, activated by both negative and positive prediction errors, are similar to those proposed to promote learning in conditions of uncertainty [109]. Studies utilizing optogenetics to stimulate dorsal raphe serotonergic neurons and pharmacological studies employing the use of SSRIs both indicate enhancement of serotonergic function will lead to enhanced learning [110,111,112].

In a similar vein, studies of serotonergic depletion result in deficits in reversal learning and impaired cognitive flexibility [113,114]. Primate studies have revealed that 5-HT depletion in the PFC impairs performance on serial discrimination reversal tasks, indicating serotonin plays a crucial role in adapting responses based on changing stimulus–reward relationships and updating task rules [115]. Thus, 5-HT depletion primarily impairs the ability to adapt attentional focus rather than change attentional strategy [116]. 

A region of the prefrontal cortex, the orbitofrontal cortex (OFC), is largely responsible for these deficits in cognitive flexibility due to 5-HT depletion. Targeted depletion of 5-HT in the OFC significantly impairs reversal learning in both rodents and marmosets [113,115,117]. This function of the PFC of being involved in cognitive flexibility can be traced back to serotonergic receptors 5-HT2A and 5-HT2C. When employing antagonist M100907 (0.1 mg/kg, IP, 20 min prior to behavioral task) to target 5-HT2A receptors during a spatial discrimination and reversal task, the respective antagonist significantly impaired reversal learning in rats. Meanwhile, employing the antagonist SB 242084 (0.3 and 1.0 mg/kg, IP, 20 min prior to behavioral task) targeting 5-HT2C receptors significantly improved reversal learning, implying the serotonergic receptors have distinct roles in cognitive flexibility and response inhibition in cases of learning [118]. Further work by Bekinschtein and colleagues additionally revealed that inhibiting 5-HT2A receptors is necessary for mPFC-hippocampal interaction during memory retrieval of a spontaneous novelty performance task, emphasizing the prefrontal-serotonergic influence in the control of memory retrieval [119]. 

Like dopamine, the serotonergic system has also been indicated in social learning. Learning through others is an important skill in order to adapt behavior and avoid harmful defeats through tracking and updating neural computations. In rodents, forms of aggressive social confrontation lead to sustained decreases in 5-HT levels to 80% of baseline levels [120]. Human depression studies reveal 5-HT depletion during a social learning task can impair social reward learning on fMRI scanning. These subjects showed altered social reward prediction signals in the insula, temporal lobe, and PFC, indicating there are both behavioral and neural impairments in learning from social rewards with 5-HT depletion [121]. Additional human studies provide further support that the PFC encodes social prediction errors, and that the computations required to learn social tasks are influenced by serotonin transporter levels [122].

### 3.3. Norepinephrine: Attention, Working Memory, and Emotional Consolidation

The cognitive ability to either switch or maintain attention can be crucial for attaining goals. Depletion of NE within the PFC impairs the ability to shift attention to a cue of a different sensory modality, supporting the notion that the norepinephrine system assists in promoting both flexible and sustained attention in the PFC to promote learning [123,124]. In rats, elevation of NE activity of the α1 receptor family in the medial prefrontal cortex is found to improve attentional set-shifting task performance [125]. Blockade of β-adrenergic receptors with propranolol in human participants impaired detection of stimuli and attention, independent of target valence [126]. Furthermore, clonidine, an α2 receptor agonist, has been tested and approved as a medication for attention-deficit hyperactivity disorder (ADHD) at a dose of 0.1–0.4 mg/day, twice a day [127]. Thus, NE and subsequent receptor subtypes serve as important mediators for attention and vigilance in the PFC.

Immunotoxic ablation of LC neurons significantly impairs working memory performance while leaving reference memory intact, indicating NE afferents are involved in more complex aspects of cognitive performance outside of arousal and attention [128]. Rodent, primate, and human studies have highlighted the valuable role of norepinephrine in functions of working memory [76]. While stimulation of the low-affinity α1 receptors is more closely linked to a stress-like impairment in working memory, the high-affinity α2 receptors are associated with improvements in working memory [124]. Systemic treatment of α2 agonists (clonidine, 0.0001–0.05 mg/kg) in primates who suffer WM impairments due to catecholamine depletion leads to improvements in WM performance [129,130]. Furthermore, α2 agonists such as clonidine (0.001–0.01 mg/kg, subcutaneously) and guanfacine (0.05–1.0 mg/kg, subcutaneously) are found to ameliorate spatial working memory deficits displayed by rats administered PCP, an NMDA/glutamate antagonist [131]. In order to orchestrate the underpinnings of working memory, activation of α2A receptors inhibits cAMP signaling and thus reduces hyperpolarization-activated cyclic nucleotide-gated channel (HCN) activation, thereby strengthening the functional connectivity of PFC networks [132]. Further investigation of NE’s role in WM has revealed that within the PFC, like other monoamines, optimal NE leads to sustained activity in pyramidal neurons via pre- and postsynaptic α1 adrenoceptors and postsynaptic α2 adrenoceptors. This mechanism may be a crucial component of working memory [76,133]. When the PFC instead receives excessive stimulation of α1 receptors from high NE levels, the result is abnormal neuronal activity causing disrupted WM capability [133,134]. Similarly, β1 adrenoceptor stimulation can further impair WM performance, while blocking β1 activity improves WM [52,76,135]. 

A longstanding hypothesis has stated norepinephrine mediates enhanced memory consolidation of emotional events [136]. NE is previously found to lower the threshold for memory formation through phosphorylation of GluR1 [137]. Within the PFC, NE is released during stress and emotional arousal and therefore may be central for the emotional regulation of memory formations [138]. Fear conditioning largely requires emotional arousal in order to enhance sensory perception and increase arousal, therefore it is not surprising that norepinephrine is necessary for fear conditioning through prefrontal synapse modulation [139,140,141]. In order to characterize norepinephrine’s role in threat prediction, Basu and colleagues obtained NE recordings during fear learning and reinforcement learning. Intervals between auditory cues and aversive stimuli were varied to determine the ability of NE to represent different timescales of threat prediction. The group found NE signaling supports the generation of threat prediction errors, and PFC-NE release is representative of the strength of the fear association to a learned cue. Interestingly, they found that during a trace period between cue and outcome, NE release decays, indicating it acts as prediction error rather than threat evaluation [53]. Optogenetic and pharmacologic manipulations of norepinephrine transmission throughout the encoding of fear memory have been found to require local activation of β-adrenergic receptors [51].

## 4. Conclusions and Future Directions

In summary, the coordinated activities of dopamine, serotonin, and norepinephrine within the PFC facilitate various aspects of learning, including attentional processing, reinforcement, synaptic plasticity, working memory, and circuit-level modulation (Table 1). While the specificity of many compounds and tools currently available is insufficient to guarantee complete translatability between species, insights gained from such studies are valuable in displaying the underlying function of monoamines in higher cognition. Working together, these neuromodulators ensure efficient encoding, consolidation, and retrieval of information, ultimately contributing to adaptive behavior and cognitive flexibility. 

The following future directions are needed to advance our understanding of monoamine function in the prefrontal cortex and its impact on cognitive processes:

***Temporal Dynamics and Longitudinal Studies:*** Future research should focus on understanding the temporal dynamics of monoamine fluctuations and their long-term changes, particularly in relation to cognitive functions. Investigating how monoamine levels and their receptor interactions evolve over time will provide critical insights into the developmental trajectories and aging processes affecting cognitive functions. This approach can also illuminate the neurobiological underpinnings of cognitive decline associated with aging and disease.

***Individual Variability:*** There is a significant gap in understanding the individual variability in monoamine systems. Exploring how genetic predispositions, environmental exposures, and lifestyle choices influence individual differences in monoamine signaling is crucial. Such studies will help clarify why certain individuals are more susceptible to cognitive disorders and aid in the development of personalized therapeutic interventions.

***Integration of Multi-Modal Data:*** Integrating multi-modal imaging data with genetic, behavioral, and clinical information could offer a more comprehensive understanding of how monoamines influence cognitive functions. This holistic approach would facilitate a deeper understanding of the complex interactions between neurobiological processes and cognitive behaviors, potentially leading to innovative strategies for cognitive enhancement and disorder management.

***Mechanisms of Monoaminergic Drugs:*** Despite the widespread use of psychoactive drugs targeting monoamine systems, the precise mechanisms by which these drugs modulate cognitive functions remain poorly understood. Future research should dissect the specific neural pathways and circuit-level interactions influenced by these drugs to enhance therapeutic efficacy and reduce side effects.

***Cross-Talk Between Different Monoamine Systems:*** Investigating the interactions between different monoamine systems (dopamine, serotonin, and norepinephrine) and their collective impact on cognitive functions is essential. Understanding how these systems modulate each other could reveal new targets for therapeutic intervention, particularly in complex neuropsychiatric conditions where multiple systems are implicated.

***Advanced Circuit Manipulation Techniques:*** The next frontier in neuroscience involves enhancing the resolution and specificity of circuit manipulation tools like optogenetics and chemogenetics, integrating them with emerging technologies for dynamic control of neuronal activity. Future innovations should focus on developing multi-wavelength optogenetic tools for simultaneous targeting of multiple neuronal populations and improving the temporal precision to match the rapid dynamics of cognitive processes. Additionally, advancements in chemogenetics need to produce faster-acting, reversible, and more bioavailable designer drugs. Integrating these tools with live-imaging technologies, such as two-photon microscopy or real-time fMRI, will bridge cellular-level interventions with whole-brain dynamics, providing real-time insights into how specific neuronal manipulations affect brain activity during behavioral tasks. Furthermore, expanding tool specificity to target neuronal compartments and developing computational models to simulate intervention effects will deepen our understanding of monoamine functions in the PFC and their roles in cognition, ultimately guiding novel therapeutic strategies for neurological disorders.

***Ethical and Clinical Translatability:*** As research in this field advances, it will be important to consider the ethical implications of manipulating brain chemistry and to ensure that findings from animal models are translatable to human clinical treatments. Addressing these issues will be crucial for the responsible development and application of new cognitive therapies.

By addressing these key areas, future research can significantly advance our understanding of monoamine function in the PFC, improving our ability to manage cognitive health and treat disorders associated with monoamine dysregulation.

## Figures and Tables

**Table 1:** Highlighted behavioral effects from selected studies. Based on the findings of the present review, we present the binding mechanism of select receptor families of DA, 5-HT, and NE alongside the behavioral output from an applied agonist or antagonist to the receptor.

Receptor Family	Binding Mechanism	Ligand	Behavior
D1-like (D1 and D5)	G_s_ coupled	Agonist	Social dominance (*mice*)
		Antagonist	Impaired social learning (*humans*)
D2-like (D2, D3, D4)	G_i/o_ coupled	Agonist	Social submission (*mice*)
5-HT2A	G_q_ coupled	Antagonist	Impaired reversal learning (*rats*)
5-HT2C	G_q_ coupled	Antagonist	Improved reversal learning (*rats*)
α1	G_q_ coupled	Agonist	(in mPFC) Improves attentional set-shifting task performance (*rats*)
α2	G_i/o_ coupled	Agonist	Ameliorates spatial working memory deficits (*rats*, *primates*)
β	G_s_ coupled	Antagonist	Impairs detection of stimuli and attention independent of target valence (*humans*) Improves working memory (*primates*)

## Data Availability

Not applicable.
